# Psychometric properties of the frequency of suicidal ideation inventory (FSII) in pregnant women: evidence from SEM, measurement invariance, and IRT models

**DOI:** 10.3389/fgwh.2026.1841080

**Published:** 2026-06-18

**Authors:** Jonatan Baños-Chaparro, Tomás Caycho-Rodríguez, Juan Artica-Martinez, Claudia Saldaña-Díaz, Miluska Moreyra-Ruiz, Armando Palacios-Rejas, Esteban Sarmiento-Suarez

**Affiliations:** 1 Facultad de Ciencias de la Salud, Universidad Privada Norbert Wiener, Programa Académico de Psicología, Lima, Peru; 2 Facultad de Psicología, Universidad Científica del Sur, Lima, Peru; 3 Departamento de Psicología, Instituto Nacional Materno Perinatal, Lima, Peru; 4 Escuela de Posgrado, Universidad Ricardo Palma, Lima, Peru

**Keywords:** mental health, pregnant women, psychometrics, risk factors, suicidal behaviors

## Abstract

**Introduction:**

Suicidal ideation during pregnancy constitutes a significant public health concern due to its serious consequences for both the mother and the fetus. Despite its relevance, the accurate assessment of this construct in pregnant women remains limited, particularly in Latin American contexts. In this regard, having psychometrically sound and culturally appropriate instruments is essential for early detection and timely intervention.

**Objective:**

To analyze the psychometric properties of the Frequency of Suicidal Ideation Inventory (FSII) in pregnant women.

**Methods:**

A total of 962 Peruvian pregnant women participated, completing a sociodemographic questionnaire, the Frequency of Suicidal Ideation Inventory (FSII), the Athens Insomnia Scale (AIS), the Patient Health Questionnaire-2 (PHQ-2), and the Generalized Anxiety Disorder-2 (GAD-2). Structural equation modeling, Bayesian reliability, and item response theory were applied for statistical analysis.

**Results:**

The FSII demonstrated adequate content validity (*V* > 0.70), a unidimensional structure [CFI = 0.99, TLI = 0.99, RMSEA = 0.07 (90% CI: 0.050, 0.099), SRMR = 0.01], and high reliability (*ω* = 0.94, *H* = 0.98). Additionally, factorial invariance was supported according to pregnancy planning status, and significant positive associations were found with depressive symptoms, generalized anxiety, and insomnia. Item 4 showed the highest discrimination and information, and the scale proved to be most precise at higher levels of suicidal ideation.

**Conclusions:**

The FSII shows adequate psychometric properties that support its factorial structure and reliability for interpreting scores in Peruvian pregnant women. Evidence of factorial invariance by pregnancy planning and its utility at higher levels of suicidal ideation further support its use in this population. Therefore, its application is recommended in both clinical practice and research, contributing to the detection and prevention of suicidal risk during pregnancy.

## Introduction

Suicidal behavior can be conceptualized as a spectrum of thoughts related to one's own death or the act of taking one's life, ranging from passive ideas to more active and structured forms of planning (1). In the perinatal context, its clinical importance lies in its role as a proximal indicator of suicide risk, allowing early identification before the occurrence of self-harm behaviors or suicide attempts (2). Epidemiological evidence suggests that, during pregnancy, suicidal ideation is not uncommon and may occur with a prevalence similar to or even higher than that observed in the general population, although with considerable variation depending on the country and the period assessed (2–4). For example, an international review reported that rates among pregnant women range approximately between 5% and 20%, reaching even higher values in certain contexts (4), while another review estimated a global range between 2.7% and 18% (2). In Latin America, the available evidence remains limited and methodologically heterogeneous; however, clinically relevant levels have already been identified both in adult populations and in particularly vulnerable groups such as adolescents (3, 5). In the case of Peru, a study conducted with 1,517 pregnant women receiving care in Lima reported prevalence rates of suicidal ideation ranging from 8.8% to 15.8%, also showing that these estimates depend on the instrument used and the reference period considered (6).

Mental health during pregnancy is an essential component of women's overall well-being and proper fetal development. However, pregnant women face multiple mental health problems, including depression, generalized anxiety, perceived stress, and insomnia (4, 7, 8). The literature has demonstrated a strong association between suicidal ideation and these factors, particularly depressive symptoms, anxiety disorders, and sleep disturbances (2, 3). From a theoretical perspective, models such as the interpersonal theory of suicide propose that suicidal ideation emerges when perceived burdensomeness and social disconnection coexist, which may intensify during pregnancy in vulnerable contexts (9). Likewise, insomnia and poor sleep quality may increase dysphoria, nighttime rumination, emotional dysregulation, and deficits in decision-making; a systematic review with meta-analysis reported more than a threefold increase in perinatal suicide risk associated with sleep disturbances (7, 10). Meanwhile, depressive symptoms and generalized anxiety contribute to a negative view of the future and a perceived inability to cope with the demands of pregnancy (2, 4).

The experience of suicidal ideation also differs depending on whether the pregnancy was planned or unplanned. Evidence suggests that unplanned pregnancies are associated with poorer maternal mental health, including a higher likelihood of depression during pregnancy and greater exposure to interpersonal violence (11). Additionally, one study reported that postpartum depression fully mediated the relationship between unplanned pregnancy and suicide risk (12). Furthermore, a systematic review and meta-analysis indicated a higher overall risk of behavioral disorders and a pooled prevalence of 65%, suggesting psychiatric vulnerability among women with unplanned pregnancies (13). These conditions increase the likelihood of suicidal ideation compared to women whose pregnancies were desired. From a theoretical perspective, the stress and coping model suggests that an unplanned pregnancy may be perceived as a stressful event that exceeds available personal resources, generating negative emotional responses (8). In addition, the theory of perceived control posits that lack of planning may reduce feelings of agency and autonomy, contributing to hopelessness and, consequently, to the emergence of suicidal thoughts (14).

The assessment of suicidal ideation in pregnant women is essential for early detection and the implementation of effective preventive interventions. Various psychological instruments are used to measure suicidal ideation, such as the Beck Scale for Suicidal Ideation (BSS), the Columbia-Suicide Severity Rating Scale (C-SSRS), the Paykel Suicide Scale (PSS), the Suicidal Behaviors Questionnaire-Revised (SBQ-R), and the Roberts Suicidal Ideation Scale (EISR) (15–19). However, many of these instruments include items on non-suicidal self-injury or present limitations in terms of length, dichotomous response formats (which reduce variability), and clinical applicability. In this context, the Frequency of Suicidal Ideation Inventory (FSII) stands out as a brief, easy-to-administer and interpret tool focused on the frequency of suicidal thoughts over the past twelve months (20). Moreover, the FSII has demonstrated solid psychometric evidence in different countries such as the United States, Turkey, China, Hungary, Brazil, Spain, and Peru (20–24). A recent meta-analysis also indicated that the FSII is a consistent and reliable instrument for research use (25). However, most adaptations have been conducted in university students (20, 24) and general adult populations (21–23). Regarding the statistical approach, most studies have used structural equation modeling (SEM) for confirmatory factor analysis and relationships with other variables, while only two studies have examined measurement invariance (21, 23).

Based on this background, an important limitation in the depth of psychometric analysis is evident, as approaches such as item response theory have not been incorporated. This framework allows examination of individual item functioning, estimation of discrimination and difficulty parameters, and evaluation of measurement precision across the latent trait continuum (26). Likewise, the absence of reliability estimates from a Bayesian perspective limits understanding of the uncertainty associated with scores and the stability of parameters, especially in contexts with specific sample sizes or particular populations (27). Together, these approaches would provide more robust and detailed evidence regarding instrument performance. Finally, there is no adaptation of the FSII for pregnant women, representing a relevant gap given the emotional and contextual particularities of this group, and highlighting the need to evaluate its psychometric properties in this population.

In this sense, the present study is relevant as it focuses on a vulnerable and understudied population in the Peruvian context. The general objective is to analyze the psychometric properties of the FSII in pregnant women. Specifically, it aims to: (a) evaluate the content validity of the instrument, (b) analyze its factorial structure using structural equation models, (c) estimate the reliability of the FSII, (d) examine measurement invariance according to pregnancy planning status, (e) analyze the relationship between the FSII and other relevant psychological variables such as depressive symptoms, generalized anxiety, and insomnia, and (f) analyze item functioning and scale precision using item response theory. These analyses allow determining the suitability of the FSII as an assessment tool in pregnant women and contribute to the development of detection and intervention strategies in maternal mental health.

## Methods

### Design

The study corresponds to an instrumental design, in which the psychometric properties of the FSII are analyzed (28). Additionally, it is a basic, cross-sectional study with a quantitative approach, aimed at examining the validity and reliability of the instrument in a sample of Peruvian pregnant women.

### Participants

A total of 962 Peruvian pregnant women participated in the study. They were selected through non-probabilistic convenience sampling and met the following inclusion criteria: (a) being 18 years of age or older, (b) residing in Metropolitan Lima, (c) receiving perinatal care at the National Maternal and Perinatal Institute, and (d) being in the first, second, or third trimester of pregnancy. The exclusion criteria were: (a) diagnosis of an intellectual or neurological developmental disorder that prevented completion of the survey, (b) incomplete responses in the sociodemographic section or in the psychological instruments, and (c) voluntary withdrawal from the survey. Sample size estimation was conducted based on the hypothetical factorial model of the FSII (5 items, one factor), considering four parameters: an expected CFI of 0.95, an average factor loading of 0.60, statistical power of 99%, and a significance level of *p* = 0.05 (29). The minimum required sample size was 611 participants. The final sample exceeded this recommendation.

### Instruments

#### Sociodemographic information

A section was created to collect participants' sociodemographic data, including: age, employment, marital status, area of residence, educational level, gestational stage, planned pregnancy, paternal rejection, suicidal ideation and suicide attempts during pregnancy, abortion history, current intention to terminate the pregnancy, and attitude toward pregnancy. Considering the Peruvian legal context, where abortion is legally restricted except in specific circumstances, questions related to abortion history and intention to terminate pregnancy were approached with strict confidentiality and ethical safeguards.

#### Frequency of suicidal ideation inventory (FSII)

This instrument measures the frequency of suicidal thoughts during the past year through five items: 1) how often have you thought about hurting yourself?, 2) how often have you believed that you did not deserve to live?, 3) how often have you wondered what would happen if you ended your life?, (4) how often have you thought about committing suicide?, 5) how often have you wished not to exist? (20). Each item is rated on a Likert scale from 1 (never) to 5 (almost always). The total score ranges from 5 to 25, with higher values reflecting a greater frequency of suicidal ideation. In this study, the version adapted for the general Peruvian adult population was used, which demonstrated adequate internal consistency (*α* = 0.95) (21).

#### Athens insomnia scale (AIS)

This scale consists of five items that assess the presence of insomnia symptoms during the past month, considering a frequency of at least three times per week. Responses are recorded on a Likert scale from 0 to 3 (30). Total scores range from 0 to 15, where higher scores indicate greater severity of insomnia symptoms. The general Peruvian adult population was used, showing adequate internal consistency (*α* = 0.89) in the analyzed sample (31).

#### Patient health questionnaire-2 (PHQ-2)

This is a brief instrument with two items that measure depressive symptoms during the past two weeks. Each item is rated on a 4-point scale from 0 (not at all) to 3 (nearly every day) (32). Total scores range from 0 to 6, where higher scores reflect greater severity of depressive symptoms. The general Peruvian adult population was used and demonstrated adequate internal consistency (*α* = 0.79) (33).

#### Generalized anxiety disorder-2 (GAD-2)

This is a brief instrument composed of two items that measure generalized anxiety symptoms during the past two weeks. Each item is rated on a 4-point scale from 0 (not at all) to 3 (nearly every day) (34). Total scores range from 0 to 6, with higher scores indicating greater levels of generalized anxiety. The general Peruvian adult population was used and demonstrated adequate internal consistency (*α* = 0.86) (35).

### Procedure

For the content validity stage, five expert judges in clinical psychology were invited to evaluate the items according to three fundamental criteria: relevance, representativeness, and clarity, with the aim of determining the adequacy of the items for measuring the construct of interest and ensuring their conceptual and semantic appropriateness within the instrument. Subsequently, prior to formal administration, a focus group with five pregnant women was conducted to assess the comprehension and clarity of the items in the target population. At this stage, no suggestions for modification were received.

Data collection was then carried out in person at the National Maternal and Perinatal Institute (NMPI), located in Cercado de Lima, during the months of October, November, and December 2025. The administration of the instruments was conducted by four psychologists working at the institution, who were previously trained to ensure proper survey administration and adherence to established ethical procedures. Additionally, the psychologists were trained to identify indicators of active suicidal ideation and to activate a predefined risk management protocol when necessary. Participants were approached in the NMPI perinatal care services, where the purpose of the study was explained and they were invited to participate voluntarily. The instruments were administered in an appropriate setting that ensured the privacy and comfort of the participants, with an approximate duration of 10–15 min.

In cases where participants reported active suicidal ideation or showed signs of psychological distress during the assessment (4 cases), an immediate intervention protocol was implemented. For risk management purposes, active suicidal ideation was operationally defined as the current presence of suicidal thoughts accompanied by indicators of increased risk, such as greater frequency or intensity of suicidal ideation, possible intent or planning, clinically significant psychological distress, or observable signs of risk during the assessment. Although 53 participants reported suicidal ideation in the general assessment, only four met the criteria for immediate intervention because they presented active suicidal ideation or significant psychological distress at the time of data collection. Participants were stratified according to risk level: those who reported suicidal ideation without indicators of immediate risk received preventive information about available mental health resources and support services, whereas those identified as having active risk were not left unattended, received immediate psychological support within the institution, were evaluated by the attending psychologist, and were referred through the established pathway to the mental health services of the NMPI for specialized assessment and care.

Prior to participation, respondents received detailed information about the objectives of the study, its exclusively academic nature, the voluntary nature of participation, data management procedures, and guarantees of anonymity and confidentiality. Written informed consent was obtained in person from all participants, in accordance with ethical standards for research involving human subjects.

Additional safeguards were implemented due to the potentially vulnerable condition of pregnant women and the sensitive nature of assessing suicidal ideation. Participants were informed that they could decline to answer any question or discontinue the survey at any time without any consequence for their clinical care or access to health services. The survey was administered by trained psychologists in a private setting, and a risk management protocol was available during data collection. If a participant experienced distress or reported active suicidal ideation, the assessment could be interrupted, immediate psychological support was provided, and referral to the NMPI mental health services was activated. Because the survey was anonymous and no contact information was linked to the research database, follow-up contact was not conducted by the research team; however, participants referred for specialized care were managed through the institutional mental health pathway, where further assessment and follow-up could be provided according to clinical procedures.

### Statistical analysis

Statistical analysis was conducted using RStudio software (version 4.3.2), following a sequence of phases for the psychometric evaluation of the FSII. In the first phase, a detailed descriptive analysis of the items was performed to assess their statistical behavior and initial quality. Measures of central tendency and dispersion (mean and standard deviation) were calculated, along with distribution shape indicators, including skewness and kurtosis. Additionally, a polychoric correlation matrix was estimated, considering the ordinal nature of the items, together with corrected item–total correlations, adopting values greater than 0.30 as the adequacy criterion (36). Furthermore, content validity was examined using Aiken's *V* coefficient, considering values above 0.70 as acceptable, in line with previous methodological recommendations (37).

In the second phase, a confirmatory factor analysis (CFA) was conducted to evaluate the internal structure of the instrument. Given the ordinal nature of the data, the weighted least squares mean and variance adjusted estimator (WLSMV) was used, as it is appropriate for this type of measurement. Model fit was assessed using several goodness-of-fit indices, including the Comparative Fit Index (CFI), Tucker–Lewis Index (TLI), Root Mean Square Error of Approximation (RMSEA), and Standardized Root Mean Square Residual (SRMR). Criteria for good fit were set as CFI and TLI values above 0.95, and RMSEA and SRMR values below 0.08 (38). Additionally, standardized factor loadings were evaluated, considering values above 0.30 as adequate (36).

In the third phase, the reliability of the instrument was estimated using the Bayesian omega coefficient (*ω*) and coefficient H (39, 40). Bayesian omega was prioritized as the main index due to its ability to provide more stable estimates and corresponding credibility intervals when working with ordinal data and moderate sample sizes (27). Unlike traditional coefficients such as ordinal alpha or classical omega, Bayesian approaches treat model parameters as random variables and incorporate prior information, improving estimation precision and reducing bias in finite samples (41). In this study, the prior distribution was set at *r* = 0.707, in accordance with simulation studies (27). In addition, model convergence was evaluated using the R-hat index, with values close to 1 indicating adequate convergence of the Markov chains, based on 1,000 iterations (41).

In the fourth phase, factorial invariance was evaluated according to pregnancy planning status using the WLSMV estimator. First, a configural model was estimated to assess the equivalence of the FSII factorial structure between planned and unplanned pregnancy groups. Subsequently, progressively more restrictive models were tested to examine equivalence at different levels: thresholds (threshold invariance), factor loadings (metric invariance), intercepts (scalar invariance), and residuals (strict invariance). Invariance was evaluated using minimal change criteria, with cut-off points of ΔCFI < 0.010, ΔTLI ≤ 0.010, and ΔSRMR ≤ 0.030 (42, 43).

In the fifth phase, a covariance-based structural equation modeling (CB-SEM) model was estimated to analyze the relationships between the study's latent variables. The WLSMV estimator was used, and overall model fit was evaluated using the CFI, TLI, RMSEA, and SRMR indices, following the same previously established criteria (38). The magnitude of relationships was interpreted according to cut-off points, considering small = 0.10, moderate = 0.30, and large = 0.50 effects (44).

Finally, in the sixth phase, a two-parameter Item Response Theory (IRT) model (2PL) was estimated. This model included the discrimination parameter (a), which reflects the item's ability to differentiate between individuals with different levels of the latent trait (*θ*), considering values greater than 1 as adequate (26), and the difficulty parameter (*β*), which represents the probability of responding to adjacent categories along the latent trait continuum. The model was specified using the Graded Response Model (GRM), and its fundamental assumptions were evaluated: unidimensionality, local independence using the LD-X^2^ statistic (LD-X^2^ < 10), and monotonicity through a nonparametric Mokken model, considering the critical statistic (crit < 0.40) (45, 46).

Model information was estimated using Item Information Curves (IIC) and the Test Information Curve (TIC). To verify proper model specification, global fit was evaluated using the C^2^ statistic, recommended for IRT models with ordinal data, along with indices such as CFI > 0.90 (47–49). Additionally, RMSEA_2_ and SRMR were considered indicators of adequate (RMSEA_2_ = 0.089, SRMR = 0.05) and excellent fit (RMSEA_2_ = 0.05, SRMR = 0.027). Since the number of response categories can influence ordinal IRT models, adjusted indices RMSEA_2_/(K − 1) and SRMR/(K − 1) were used (50). Finally, local item fit was examined using the S-X^2^ index and RMSEA.S-X^2^ < 0.06 (51, 52), to strengthen the evaluation of item-level fit.

## Ethical considerations

The study adhered to the ethical guidelines of the American Psychological Association (APA) and the Code of Ethics of the Peruvian College of Psychologists (CPsP), as described in Chapter Six on good research practices (53, 54). All pregnant women provided informed consent prior to participation. The survey was anonymous and voluntary, and confidentiality of the data was guaranteed. Given the sensitivity of questions related to abortion history and current intention to terminate pregnancy, additional confidentiality safeguards were implemented. Participants were informed during the consent process that their responses were anonymous, would be used only for research purposes, and would not affect their clinical care or access to health services. No identifying information was linked to the survey responses, and the database was de-identified before analysis. Data were stored in a password-protected digital file accessible only to the research team. The study was reviewed and approved by the Ethics Committee of Universidad Privada Norbert Wiener under registration number A0019-2025-CIEIC-UPNW (approved on June 4, 2025), as well as by the Ethics Committee of the National Maternal Perinatal Institute under registration N°0171-2025-DG-N°0118-OEAIDE-INMP (approved on September 29, 2025).

## Results

### Sociodemographic characteristics of the participants

Table 1 presents the sociodemographic characteristics of the participants. The mean age was 30.97 years (SD = 6.68), ranging from 18 to 48 years. Regarding employment status, 66.6% reported not currently working, and in terms of marital status, 63.7% were single, followed by married women (34.3%). Most participants resided in urban areas (85.3%) and had completed secondary education (34.8%), followed by technical or university education (52.5%). For international readers, technical education refers to non-university vocational training, whereas university education refers to professional degree programs typical of the Latin American educational system. Regarding gestational stage, 42.6% were in the third trimester, followed by 37.8% in the second trimester and 19.5% in the first trimester. A total of 50.2% reported that the pregnancy was planned, and 76.5% indicated absence of paternal rejection. Additionally, 5.5% reported suicidal ideation and 1.1% reported having attempted suicide. Regarding abortion history, 53.2% indicated having had none, although 37.6% reported a miscarriage. Finally, 96.6% reported no current intention to terminate the pregnancy, and 52.3% expressed a positive attitude toward it.

**Table 1:** Sociodemographic characteristics of the participants.

Variable	*n* (%)
Age	30.97 ± 6.68
Employment	
Yes	321 (33.4%)
No	641 (66.6%)
Marital status	
Single	613 (63.7%)
Married	330 (34.3%)
Divorced	15 (1.6%)
Widowed	4 (0.4%)
Area of residence	
Urban	821 (85.3%)
Rural	141 (14.7%)
Education level	
No formal/primary education	23 (2.4%)
Secondary education	429 (44.6%)
Technical education	233 (24.2%)
University education	266 (27.7%)
Postgraduate education	11 (1.1%)
Gestational stage	
First trimester (1–3 months)	188 (19.5%)
Second trimester (4–6 months)	364 (37.8%)
Third trimester (7+ months)	410 (42.6%)
Planned pregnancy	
Yes	483 (50.2%)
No	479 (49.8%)
Paternal rejection	
Never	736 (76.5%)
Rarely	131 (13.6%)
Sometimes	59 (6.1%)
Often	18 (1.9%)
Almost every day	18 (1.9%)
Suicidal ideation	
Yes	53 (5.5%)
No	909 (94.5%)
Suicide attempt	
Yes	11 (1.1%)
No	951 (98.9%)
History of abortion	
None	512 (53.2%)
Spontaneous abortion	362 (37.6%)
Therapeutic induced abortion	41 (4.3%)
Voluntary induced abortion	47 (4.9%)
Current abortion intention	
Yes	33 (3.4%)
No	929 (96.6%)
Attitude toward pregnancy	
Very negative	10 (1%)
Negative	12 (1.2%)
Neutral	131 (13.6%)
Positive	503 (52.3%)
Very positive	306 (31.8%)

### Evidence based on content

Expert judges evaluated the content of the items according to the criteria of relevance, representativeness, and clarity, obtaining Aiken's *V* values above 0.70 (Table 2). All expert judges (*n* = 5) approved the final version of the FSII. Likewise, in the focus group sample (*n* = 5), no suggestions or modifications were reported.

**Table 2:** Content validity of the FSII items.

Items	Relevance (*n* = 5)		Representativeness (*n* = 5)		Clarity (*n* = 5)	
	*V*	CI 95%	*V*	CI 95%	*V*	CI 95%
1	0.83	0.65, 0.93	0.83	0.65, 0.93	1.00	0.87, 1.00
2	0.87	0.67, 0.95	0.93	0.75, 0.99	0.83	0.65, 0.93
3	1.00	0.87, 1.00	0.87	0.67, 0.95	0.93	0.75, 0.99
4	0.93	0.75, 0.99	0.83	0.65, 0.93	1.00	0.87, 1.00
5	0.83	0.65, 0.93	0.93	0.75, 0.99	0.87	0.67, 0.95

*V*, Aiken's *V*; CI, confidence intervals.

### Descriptive analysis

Table 3 shows that the highest arithmetic mean was observed for item 3 (*M* = 1.30), while the lowest corresponded to item 4 (*M* = 1.20). Regarding variability, the largest standard deviation was found for item 5 (SD = 0.74), and the smallest for item 1 (SD = 0.59). Skewness and kurtosis values revealed substantial deviations from normality, with high positive skewness (*g*_1_ > 2) and high kurtosis (*g*_2_ > 6) across all items. These results indicate distributions skewed toward lower values, suggesting a low frequency of suicidal ideation in the evaluated sample. Likewise, the high kurtosis reflects a concentration of responses in the lower categories of the scale. On the other hand, all corrected item–total correlations were satisfactory, exceeding the 0.30 criterion. Additionally, the polychoric correlation matrix showed positive relationships among items, with no evidence of multicollinearity (*r* > 0.95).

**Table 3:** Descriptive measures and correlation matrix.

Item	*M*	SD	*g*_1_	*g*_2_	*r*_it_	Polychoric correlation matrix				
1	1.21	0.59	3.07	9.64	0.85	–				
2	1.28	0.70	2.74	7.61	0.83	0.89	–			
3	1.30	0.73	2.59	6.30	0.83	0.87	0.89	–		
4	1.20	0.65	3.58	12.78	0.88	0.94	0.90	0.90	–	
5	1.28	0.74	2.95	8.67	0.87	0.90	0.90	0.87	0.93	–

*M*, means; SD, standard deviation; *g*_1_, skewness; *g*_2_, kurtosis; *r*_it_, corrected item test correlation.

### Evidence based on internal structure

**Figure 1:**
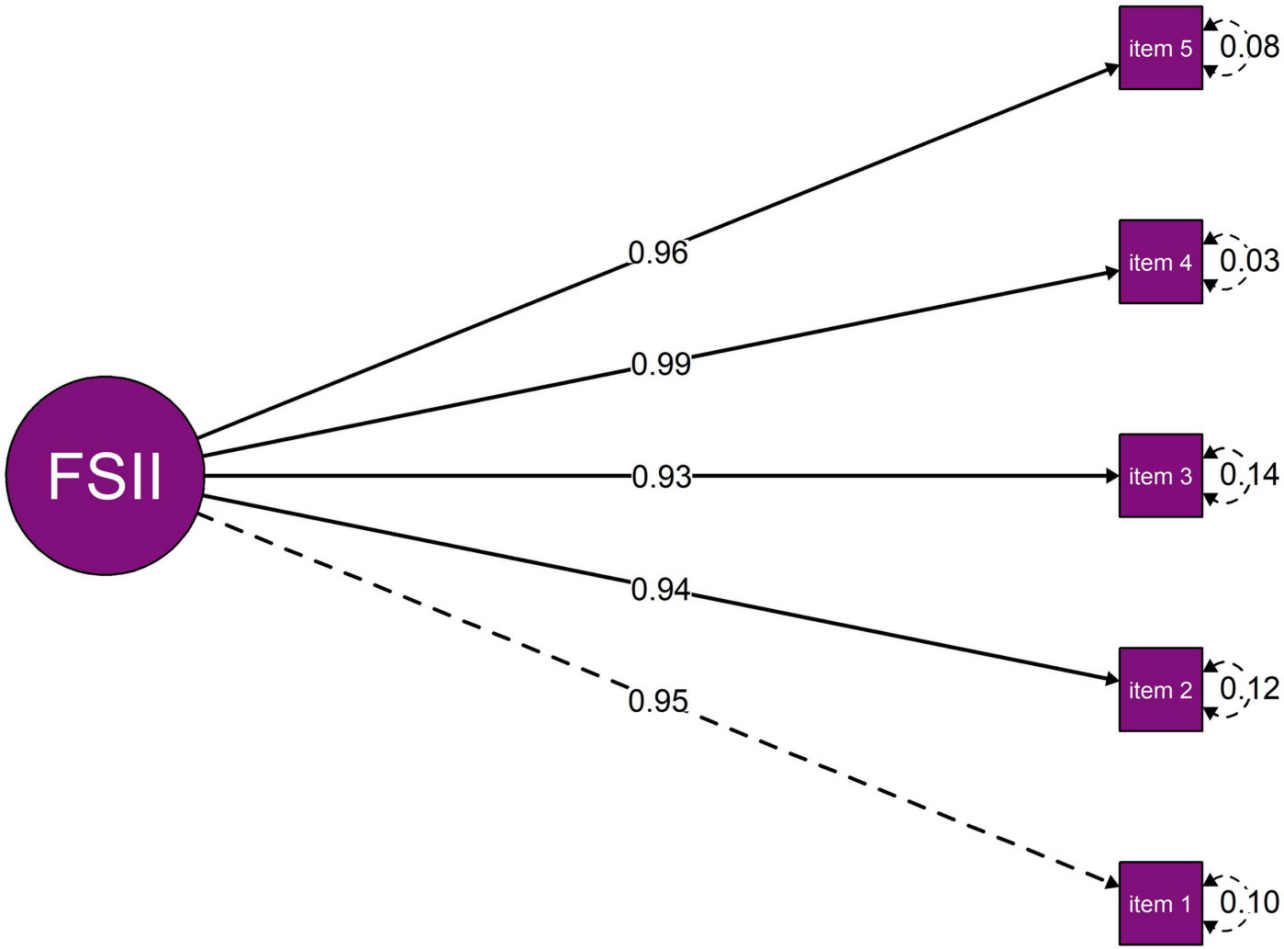
Factor structure of the FSII. Solid lines represent standardized factor loadings, whereas dashed lines represent measurement error variances.

The confirmatory factor analysis supported a unidimensional structure of the FSII, showing an overall acceptable model fit [CFI = 0.99, TLI = 0.99, RMSEA = 0.07 (90% CI: 0.050, 0.099), SRMR = 0.01]. Although the CFI, TLI, and SRMR values indicated very good fit, the upper bound of the RMSEA confidence interval approached values commonly interpreted as less favorable. Therefore, the CFA results should be interpreted as supporting the proposed unidimensional structure, but with some caution regarding the RMSEA estimate. Standardised factor loadings ranged from 0.93 to 0.99, indicating that all items contributed meaningfully to the latent construct (Figure 1).

### Reliability

**Figure 2:**
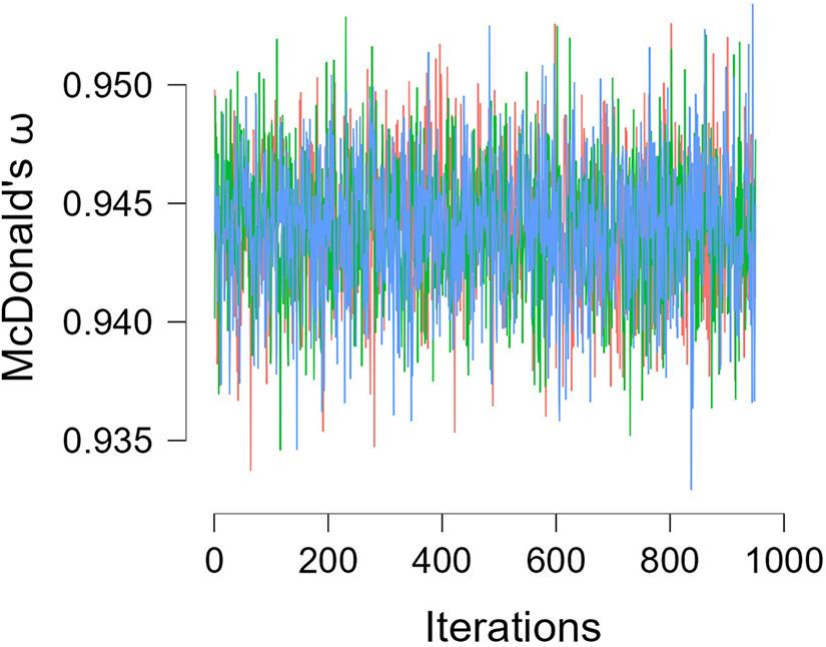
FSII convergence traceplot.

The posterior estimate of the *ω* coefficient was 0.94, with a 95% credibility interval ranging from 0.93 to 0.95, indicating a 95% probability that the true value of *ω* lies within this range. In addition, it showed adequate convergence, with Rhat = 1.000 (Figure 2). Likewise, the reliability coefficient H was 0.98, further supporting the internal consistency of the FSII.

### Measurement invariance

Table 4 presents the results of the factorial invariance analysis. It is observed that the configural model does not differ significantly from the more restrictive models—those imposing constraints on factor loadings, thresholds, intercepts, and residuals—since the changes in fit indices (ΔCFI < 0.010; ΔTLI ≤ 0.010; ΔSRMR < 0.030) remain within acceptable limits. These results support the invariance of the FSII across planned (*n* = 483) and unplanned (*n* = 479) pregnancy groups. Because scalar invariance was achieved, the latent means of both groups were compared, showing statistically significant differences with a small effect size [*t*(df) = 4.80 (960), *p* = 0.001, *d* = 0.31], with suicidal ideation scores being higher in the unplanned pregnancy group (*M* = 6.76) than in the planned pregnancy group *M* = 5.81).

**Table 4:** FSII invariance analysis according to pregnancy planning status.

Models	*X*^2^ (df)	*p*	CFI	TLI	SRMR	ΔCFI	ΔTLI	ΔSRMR
M1	30.970 (10)	0.001	0.999	0.998	0.012	–	–	–
M2	30.773 (20)	0.058	0.999	0.999	0.012	0.000	0.001	0.000
M3	31.789 (24)	0.132	0.999	0.999	0.012	0.000	0.000	0.000
M4	32.651 (28)	0.248	0.999	0.999	0.012	0.000	0.000	0.000
M5	34.564 (33)	0.393	0.999	0.999	0.013	0.000	0.000	0.001

M1, configural invariance, M2, threshold invariance, M3, metric invariance, M4, scalar invariance, M5, strict invariance.

### Evidence based on the relationship with other variables

**Figure 3:**
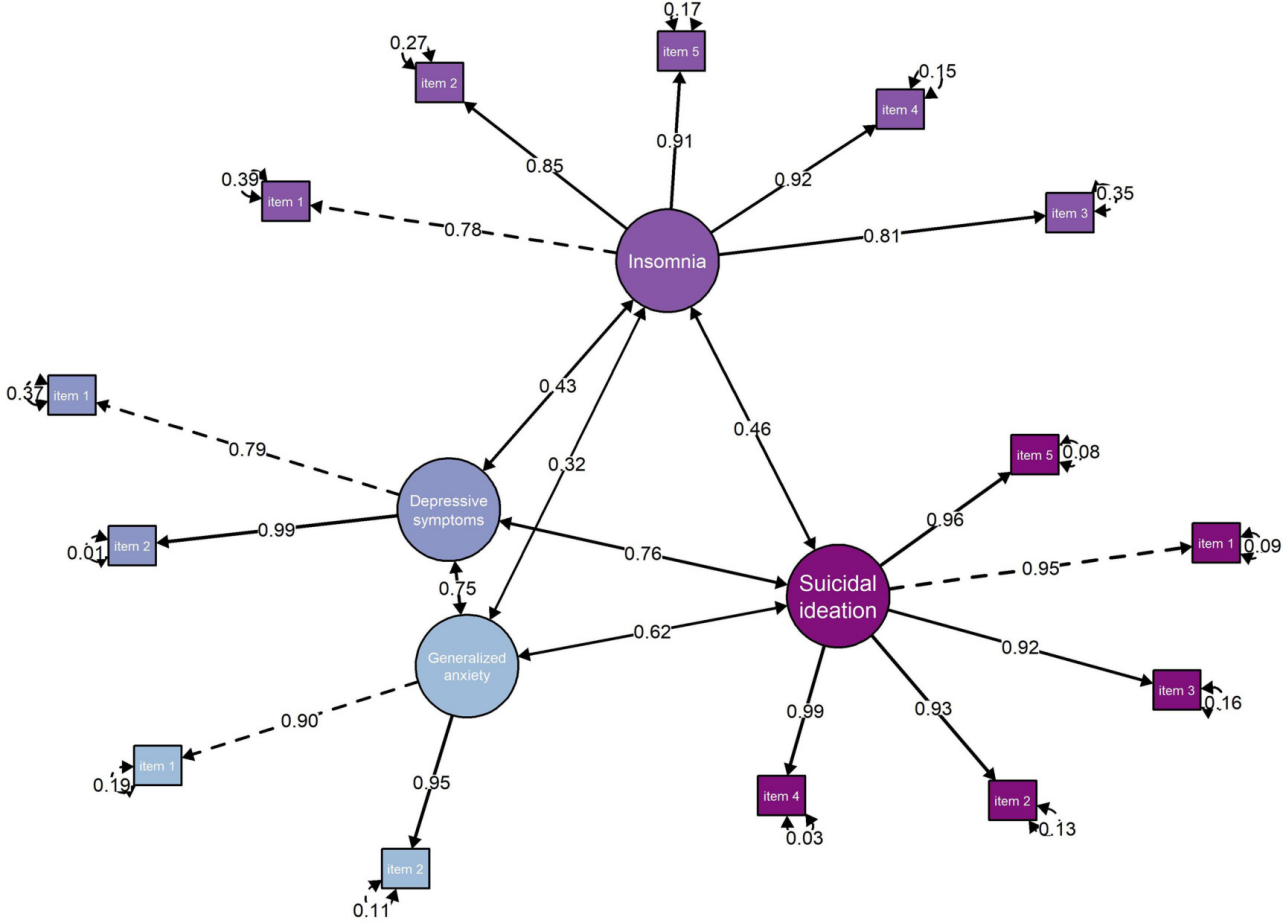
Structural model of the relationship between suicidal ideation, insomnia, depressive symptoms, and generalized anxiety in pregnant women. Solid lines indicate standardized regression paths between latent variables and factor loadings of the observed indicators, whereas dashed lines represent measurement error variances.

Figure 3 illustrates the results of the correlation analysis examining the associations between suicidal ideation and the psychological variables included in the model. The estimated model showed an adequate fit to the data: CFI = 0.99, TLI = 0.99, RMSEA = 0.05 [90% CI: 0.046, 0.060], and SRMR = 0.03, supporting the robustness of the proposed relational structure.

The results indicated that suicidal ideation was positively and statistically significantly associated with all the psychological variables evaluated. In particular, large associations were observed with depressive symptoms (*r* = 0.76, *p* = 0.001) and generalized anxiety (*r* = 0.62, *p* = 0.001), while a moderate association was identified with insomnia (*r* = 0.46, *p* = 0.001).

### Item response theory

Model assumptions were evaluated and examined. Unidimensionality was supported by the CFA, while local independence was confirmed using the LD-X^2^ index, with standardized associations below 10 (range = −0.264 to 0.243). Regarding monotonicity, no significant violations were identified (crit < 0.40). At the global level, the model showed adequate fit indices [*C*^2^ = 22.02, df = 5, RMSEA_2_ = 0.05 (90% CI: 0.035, 0.085), CFI = 0.99, SRMR = 0.01].

To further support these findings, item-level fit was examined in Table 5, where the values indicated excellent fit (RMSEA.S-X^2^ < 0.06). Regarding the discrimination parameter (*a*), item 4 showed the highest ability to differentiate levels of the latent trait. Concerning the difficulty parameter (*β*), a progressive increase was observed in the probability of selecting higher response categories as the level of *θ* increased, as detailed in Table 5.

**Table 5:** Discrimination, difficulty, and item fit parameters.

Items	Item parameters					Item fit	
	*a*	*β*_1_	*β*_2_	*β*_3_	*β*_4_	*S*-x^2^ (*gl*)	*RMSEA.S-x^2^*
1	5.32	1.117	1.638	2.404	3.031	11.535 (9)	0.017
2	4.99	0.994	1.474	2.314	2.699	7.053 (8)	0.001
3	4.35	0.965	1.455	2.100	2.886	19.269 (9)	0.034
4	9.07	1.214	1.603	2.011	2.603	8.390 (9)	0.001
5	6.15	0.997	1.465	2.013	2.475	1.911 (7)	0.001

*a*, discrimination parameter; *β*, difficulty parameter; S-X^2^, fit index; df, degrees of freedom. RMSEA.S-X^2^, root mean square error of approximation.

**Figure 4:**
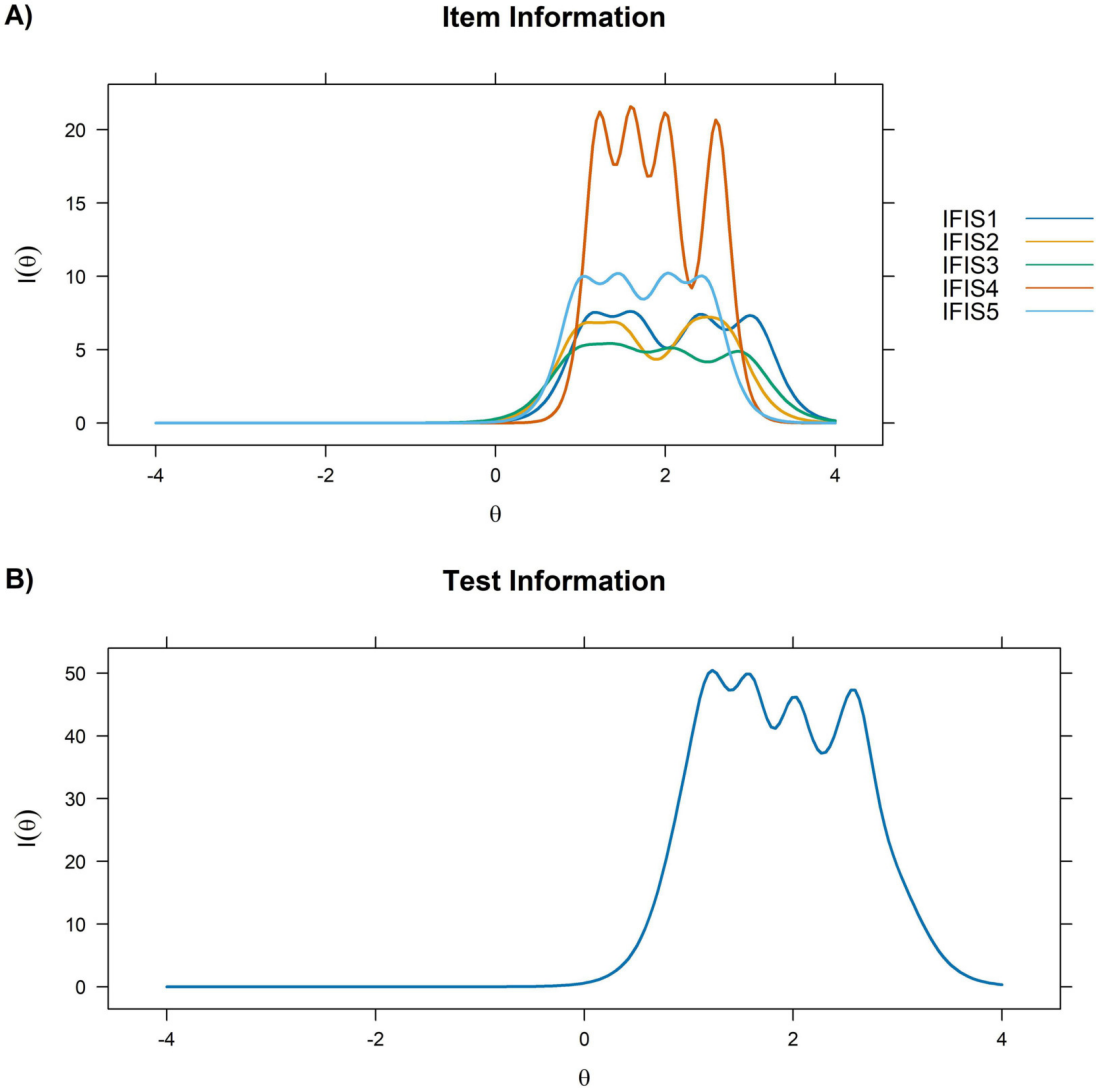
Item information function (section **A**) and test information function (section **B**) of the FSII. In Section A, each colored line represents the information provided by a specific FSII item across levels of the latent trait (*θ*). IFIS1–IFIS5 correspond to the five FSII items. In Section **B**, the solid line represents the total test information function, indicating the precision of the scale across levels of suicidal ideation.

Figure 4 (panel A) shows that item 4 provided the greatest amount of information about *θ*. Additionally, the scale is particularly precise in assessing high levels of suicidal ideation in pregnant women (Figure 4, panel B).

## Discussion

Suicidal ideation during pregnancy constitutes a relevant public health issue due to its association with adverse outcomes for both the mother and the fetus, as well as its role as a proximal indicator of suicide risk (2, 4). In this context, the availability of psychometrically sound instruments is essential for early detection and the implementation of preventive interventions. However, evidence on the assessment of suicidal ideation in pregnant women remains limited, particularly in Latin American contexts. In response to this need, the present study aimed to analyze the psychometric properties of the FSII in this population, providing evidence from multiple analytical approaches.

Regarding internal structure, the results supported a unidimensional model of the FSII, consistent with previous studies conducted in university and general adult populations (20–24). This consistency suggests that suicidal ideation, conceptualized as the frequency of thoughts related to one's own death, can be adequately represented as a unidimensional construct, even in specific populations such as pregnant women (20). From an applied perspective, this structure facilitates the clinical interpretation of the instrument, allowing for a direct and parsimonious assessment of suicidal ideation, which is particularly relevant in primary care settings where brief and efficient tools are required (2).

Regarding reliability, the results showed high levels in both the Bayesian omega coefficient and coefficient H, consistent with previous literature reporting high internal consistency of the FSII (20–24). Notably, the use of a Bayesian approach represents an important methodological strength, as it allows estimation of the uncertainty associated with reliability through credibility intervals, providing a more informative probabilistic interpretation than classical approaches (27). This type of estimation is particularly useful in applied health research contexts, where measurement precision has direct implications for clinical decision-making (39).

Regarding measurement invariance, the results indicated that the FSII is invariant between women with planned and unplanned pregnancies, implying that the instrument measures the construct equivalently across both groups. This finding extends previous evidence, as studies in Spain and Peru had demonstrated FSII invariance by sex and age groups in general adult populations (21, 23), but not in relation to specific contextual variables such as pregnancy planning. Evidence of invariance in this study is particularly relevant, given that unplanned pregnancy has been associated with greater psychological vulnerability (11, 13). Therefore, these results ensure that comparisons between groups reflect true differences in suicidal ideation rather than measurement bias.

Regarding validity based on relationships with other variables, positive and significant associations were found between suicidal ideation and depressive symptoms, generalized anxiety, and insomnia, consistent with previous literature (3, 4, 7). These associations can be explained through different theoretical frameworks. For example, the interpersonal theory of suicide proposes that negative emotional states, such as depression and anxiety, increase perceived burdensomeness and social disconnection, facilitating the emergence of suicidal ideation (9). Likewise, insomnia may exacerbate emotional dysregulation and cognitive rumination, increasing vulnerability to suicidal thoughts (10). In the context of pregnancy, these factors may intensify due to hormonal changes, psychosocial stress, and adaptive demands inherent to this stage (2, 8).

From an IRT perspective, the results showed that item 4 (“how often have you thought about committing suicide?”) had the highest discrimination and information, indicating that its content is particularly sensitive for differentiating levels of the latent trait. However, item 4 should not be interpreted in isolation, but rather as part of the total FSII score and within a broader clinical evaluation (8). Additionally, the FSII showed greater precision at higher levels of suicidal ideation, indicating that the scale is not only suitable for research purposes but also as a support tool in clinical practice, particularly in pregnant populations where early risk identification is crucial (6, 11). It is noteworthy that, unlike previous psychometric studies of the FSII, which have primarily focused on factorial analyses, the present study incorporates IRT, providing more detailed evidence on item functioning and scale precision (46, 51).

It is also important to consider the influence of the severe floor effects observed in the FSII items. The low item means, together with high positive skewness and kurtosis, indicate a strong concentration of responses in the lower categories of the scale, which is expected when assessing low-frequency but clinically relevant phenomena such as suicidal ideation in community or prenatal care samples. From a CFA perspective, these floor effects may reduce response variability and affect the estimation of thresholds, factor loadings, and standard errors, although the use of polychoric correlations and the WLSMV estimator partially mitigates this limitation when modeling ordinal and non-normally distributed data (36). From an IRT perspective, the concentration of responses in the lowest categories may contribute to item parameters that are especially informative at moderate-to-high levels of the latent trait, while providing less precision at very low levels of suicidal ideation (26). Therefore, the FSII should be interpreted primarily as a tool for identifying clinically relevant elevations in suicidal ideation rather than for finely differentiating among individuals with very low levels of the construct. Clinically, low total scores should not be interpreted as definitive absence of risk, particularly in pregnant women, and any endorsement of higher response categories should be followed by a more comprehensive suicide risk assessment.

In terms of theoretical implications, the study findings reinforce the conceptualization of suicidal ideation as a unidimensional construct that can be consistently measured across different psychometric approaches (20). Moreover, the convergence of results obtained through SEM and IRT supports the robustness of the construct and its latent representation (51). In addition, the IRT findings provide clinically meaningful information regarding item functioning and test precision, reinforcing the usefulness of the FSII for identifying clinically relevant elevations in suicidal ideation. From a practical perspective, the FSII emerges as a brief, valid, and reliable tool for assessing suicidal ideation in pregnant women, which is particularly relevant in the Peruvian context, where mental health resources are limited and early detection is key for suicide prevention (6). The distribution of item information across the latent continuum suggests that the FSII is particularly informative at moderate to high levels of suicidal ideation. In general prenatal populations, where most women are expected to report low symptom levels, this finding suggests that the FSII may be more useful for identifying clinically relevant elevations or alert cases than for finely differentiating among women with minimal suicidal ideation. Its use could support timely referral, monitoring of at-risk cases, and prioritization of preventive interventions in prenatal care services. Its systematic use could contribute to the generation of local epidemiological data on suicidal ideation in pregnant women, strengthening the planning of evidence-based public policies and maternal mental health programs.

Among the strengths of the study are the large sample size, the integration of advanced psychometric approaches such as SEM and IRT, and the incorporation of Bayesian reliability estimates, which overcome limitations of previous studies and provide new psychometric evidence for the FSII. However, some limitations should also be considered. First, the use of non-probabilistic sampling limits the generalizability of the results; specifically, the use of convenience sampling may introduce selection bias, as participants were not randomly selected and may not adequately represent the broader target population; therefore, future studies are encouraged to employ probabilistic sampling strategies to improve representativeness and extrapolation to the target population. Second, the cross-sectional design prevents assessment of the temporal stability of the instrument; more specifically, the study did not include an evaluation of test–retest reliability, which limits the ability to determine the consistency of the scores over time; thus, longitudinal studies are recommended to examine score consistency over time and provide explicit evidence of temporal stability and longitudinal validity. Additionally, the sample included a higher proportion of participants from urban areas (85.3%), which may introduce representativeness bias; this overrepresentation of urban participants limits the external validity of the findings, particularly for populations in rural settings, where contextual, socioeconomic, and healthcare access factors may differ; consequently, future studies should include more diverse samples incorporating participants from rural contexts and different geographical regions to ensure greater population heterogeneity and improve the applicability of the results across diverse settings. Another limitation is the absence of criterion validity evidence, as no gold-standard clinical assessment or structured diagnostic interview was available. Consequently, the diagnostic or screening performance of the FSII remains unknown, and sensitivity, specificity, and optimal cut-off points could not be estimated. Future studies should include clinical assessment data to determine the criterion validity of the FSII and establish clinically useful cut-off scores for prenatal care settings. Another limitation is the presence of severe floor effects across FSII items, which reflects the low frequency of suicidal ideation in the sample but may restrict score variability and limit the precision of the instrument at very low levels of the latent trait. Future studies should examine the performance of the FSII in clinical or high-risk samples, where a wider distribution of suicidal ideation scores may allow a more detailed evaluation of CFA and IRT parameter stability. Finally, other sources of validity, particularly those derived from longitudinal designs, such as test–retest reliability, were not evaluated; therefore, future research should incorporate such analyses to complement psychometric evidence support the longitudinal validation of the instrument and strengthen the stability of the instrument under different conditions. In addition, the study primarily assessed convergent validity through correlations with related constructs (e.g., depression, anxiety, and insomnia), rather than full criterion validity; no comparisons were made with established gold standard diagnostic instruments. Therefore, future research should include such reference measures to provide stronger evidence of criterion validity and enhance the clinical applicability of the instrument.

In conclusion, the present study provides robust evidence on the psychometric properties of the FSII in pregnant women from Metropolitan Lima, Peru, supporting its unidimensional structure, reliability, measurement invariance, convergent validity, and adequate item functioning. These findings support its use as a valid and reliable tool for assessing suicidal ideation in this population, contributing to the advancement of research and clinical practice in maternal mental health. Furthermore, the incorporation of complementary approaches such as SEM and IRT allows for a more comprehensive understanding of the construct, enhancing measurement precision and item-level analysis. Overall, these results not only expand the available psychometric evidence in Latin American contexts but also reinforce the relevance of the FSII as a useful instrument for the early detection of suicide risk in pregnant women. Thus, its application may contribute to the implementation of more effective, evidence-based preventive strategies, promoting timely and comprehensive care in the field of perinatal mental health.

## Data Availability

The raw data supporting the conclusions of this article will be made available by the authors, without undue reservation.
